# Interval State Estimation in Active Distribution Systems Considering Multiple Uncertainties

**DOI:** 10.3390/s21144644

**Published:** 2021-07-06

**Authors:** Tengpeng Chen, He Ren, Gehan A. J. Amaratunga

**Affiliations:** 1 Department of Instrumental and Electrical Engineering, Xiamen University, Xiamen 361102, China; tpchen@xmu.edu.cn; 2 Department of Engineering, University of Cambridge, Cambridge CB3 0FA, UK; ga@eng.cam.ac.uk

**Keywords:** interval state estimation, multiple uncertainties, distributed generation, modified Krawczyk-operator, interval constraint-propagation

## Abstract

Distribution system state estimation (DSSE) plays a significant role for the system operation management and control. Due to the multiple uncertainties caused by the non-Gaussian measurement noise, inaccurate line parameters, stochastic power outputs of distributed generations (DG), and plug-in electric vehicles (EV) in distribution systems, the existing interval state estimation (ISE) approaches for DSSE provide fairly conservative estimation results. In this paper, a new ISE model is proposed for distribution systems where the multiple uncertainties mentioned above are well considered and accurately established. Moreover, a modified Krawczyk-operator (MKO) in conjunction with interval constraint-propagation (ICP) algorithm is proposed to solve the ISE problem and efficiently provides better estimation results with less conservativeness. Simulation results carried out on the IEEE 33-bus, 69-bus, and 123-bus distribution systems show that the our proposed algorithm can provide tighter upper and lower bounds of state estimation results than the existing approaches such as the ICP, Krawczyk-Moore ICP(KM-ICP), Hansen, and MKO.

## 1. Introduction

The distribution system state estimation (DSSE) continuously utilizes the raw data measured by the supervisory control and data acquisition (SCADA) or the phasor measurement unit (PMU) to acquire the system state variables for the system controller [1,2,3,4]. With the widespread application of distributed generation (DG) and electric vehicle (EV) in the distribution systems, the DSSE would contain more significant uncertainties caused by the stochastic power outputs of DG and the plug-in EV [5,6,7]. In addition, the effects of other uncertainties generated by the inaccurate line parameters and the non-Gaussian measurement noise statistics can also be non-negligible in DSSE [8,9,10]. Many researchers propose the interval state estimation (ISE) methods to model and analyze distribution systems with multiple uncertainties [8,11,12]. The work in [11] uses different estimators to analyze the sensitivity of the uncertainty interval state estimation model. A robust interval state estimation algorithm is proposed to eliminate the assumption of specific probability density function (PDF) for the measurement error of smart meters in [12]. The work in [8] proposes an interval algorithm to calculate the unbalanced distribution system model with some uncertainties. However, one possible shortcoming of the above methods is that the uncertainties are considered partially.

The ISE model that can truly represent the characteristics of distribution systems is of great significance to DSSE [13,14]. Note that most of models utilized in DSSE are similar to those of transmission system and the bus voltage or branch current are taken as the state variables [15,16]. In addition, the branch injection power is taken as the state variables so as to reduce the influence of bad data on the state estimation results [16]. One possible disadvantage of these models is that the line parameters and power sources are assumed to be consistent. However, the utilized parameters may be affected by the complex working environment and some unavailable equipments, and then some errors would be generated [17,18,19]. Therefore, the authors of [17] establish a model considering the line parameter uncertainty and measurement uncertainty, which aims to analyze the influence of network parameter uncertainty on DSSE. The authors of [11] propose an ISE model including line parameter uncertainties and analyzes the influence of line parameters in multiple state estimators on the state estimation results. However, the ISE models mentioned above are expressed in the form of interval number, which would make the model parameters inaccurate.

Considering that a great amount of DGs and EVs are currently incorporated into the distribution systems, the uncertainties caused by the DG and EV should be well described in the ISE model [20]. The work in [21] obtains the probability density function (PDF) of DG based on the historical statistical data to characterize its uncertainty and shows that the distribution of the DG behavior is non-Gaussian. The work in [22] establishes a Gaussian mixture model that can represent any PDF, such as the PDF of DG and EV. Nevertheless, these ISE models usually assume that DG and EV obey a Gaussian distribution, which is not always true in reality. As the inaccurate settings of multiple uncertainties would lead to inaccurate state estimation results, the work in [7] uses a non-Gaussian estimator to solve the DSSE problem considering uncertainty. In comparison, the interval number model is used to describe state estimation model with uncertainties, which reduces the calculation time and increases the calculation efficiency.

Many algorithms are proposed for the distribution systems to solve the upper and lower bound of state estimation variables. For example, the Hansen algorithm presented in [23] and the Krawczyk operator algorithm proposed in [24] are commonly used in solving the ISE problem. Note that the Krawczyk operator algorithm is commonly to be used to solve the linear ISE problem. When the measurements are collected from the PMUs and SCADA at the meantime, the measurement model becomes nonlinear and another ISE based on the Krawczyk operator is presented in [24]. The authors of [8] propose a modified Krawczyk-operator (MKO) algorithm to solve the ISE problem and the state estimation results obtained by the MKO algorithm are more accurate than the solutions obtained by the Krawczyk operator algorithm. One shortcoming of the approaches mentioned above is that they cannot reduce the conservativeness of the state estimation results. The interval constraint-propagation (ICP) [14] is another algorithm that can solve the ISE problem. Considering that the ICP algorithm relies heavily on the setting of the initial value, the work in [25] proposes a KM-ICP algorithm consisting of the Krawczyk–Moore (KM) [23] and the ICP to select a useful initial value to reduce the conservatism of the state estimation results. Perhaps one disadvantage is that the introduction of DG and EV would not only aggravate the conservativeness of the state estimation results, but also reduces the computational accuracy of the above algorithms.

To the best of our knowledge, the existing ISE models only consider some uncertainties and the existing ISE solving approaches are still too conservative. Therefore, this paper establishes an ISE model including the uncertainties caused by the non-Gaussian measurement noise, the inaccurate line parameters, the stochastic power outputs of distributed generations (DG), and the plug-in electric vehicles (EV) in distribution systems, and then proposes a new ISE approach to solve the ISE problem to obtain tighter upper and lower bounds of state estimation results. The main contributions of this paper are as follows:

- A more accurate ISE model including the multiple uncertainties caused by the the non-Gaussian measurement noise, inaccurate line parameters, stochastic power outputs of DG, and plug-in EV are proposed for DSSE. To the best of our knowledge, there is no existing ISE model that includes such kind of uncertainties completely.
- A new ISE algorithm based on the MKO and ICP is proposed for the distribution systems to deal with the multiple uncertainties mentioned above and reduce the conservativeness of state estimation results.
- The proposed algorithm can obtain more tighter upper and lower bounds of state estimation results than other existing methods such as the Hansen, Krawczyk, KM-ICP, MKO, and ICP algorithms.

The rest of paper is organized as follows. The traditional state estimation model is expounded in Section 2. The proposed ISE model and algorithm are presented in Section 3. Simulations on the IEEE 33-bus, 69-bus, and 123-bus systems are introduced in Section 4. The conclusion is drawn in Section 5.

## 2. Distribution System State Estimation Model

### 2.1. Measurement Model

The measurement model used in distribution system state estimation is given by [2,26]

(1) $$\begin{array}{c} z=h(x)+e \end{array}$$

where

$$\begin{array}{ccc} z & = & {\left[z_{1},z_{2},\ldots ,z_{m}\right]}^{T}\\ x & = & {\left[x_{1},x_{2},\ldots ,x_{n}\right]}^{T}\\ h(x) & = & {\left[h_{1}\left(x\right),h_{2}\left(x\right),\ldots ,h_{m}\left(x\right)\right]}^{T}\\ e & = & {\left[e_{1},e_{2},\ldots ,e_{m}\right]}^{T} \end{array}$$

in which *z* is the measurement vector and *x* is the state vector. h(x) is the nonlinear measurement function relating *z* to *x*. *e* represents the measurement error.

The weighted least squares (WLS) estimator is widely utilized in DSSE [2]. The state estimation vector $\hat{x}$ can be obtained by minimizing the weighted sum of the squares of the measurement residuals

(2) $$\begin{array}{c} J\left(x\right)={\left[z-h\left(x\right)\right]}^{T}W\left[z-h\left(x\right)\right]={\left[z-h\left(x\right)\right]}^{T}R^{-1}\left[z-h\left(x\right)\right] \end{array}$$

where $R=\mathrm{diag}(\sigma_{1}^{2},\ldots ,\sigma_{m}^{2})$ is a covariance matrix related to the measurement noise and $W=R^{-1}$ is the weighting matrix. Let the partial derivative of the function with respect to *x* be equal to 0, that is,

(3) $$\begin{array}{ccc} \;\partial J(x)/\partial x & = & H^{T}\left(x\right)R^{-1}\left[z-h\left(x\right)\right]=0 \end{array}$$

(4) $$\begin{array}{ccc} H^{T}\left(x\right)R^{-1}H\left(x\right)\Delta x & = & H^{T}\left(x\right)R^{-1}\left[z-h\left(x\right)\right] \end{array}$$

where H(x)=∂h(x)/∂x is the Jacobian matrix. In order to make the solution $\hat{x}$ more precisely, an iteration formula to obtain $\hat{x}$ is given as

(5) $$\begin{array}{ccc} {\hat{x}}^{k+1} & = & {\hat{x}}^{k}+\Delta {\hat{x}}^{k}\\ & = & {\hat{x}}^{k}+{\left[\Pi \left({\hat{x}}^{k}\right)\right]}^{-1}H^{T}\left({\hat{x}}^{k}\right)R^{-1}\left[z-h\left({\hat{x}}^{k}\right)\right] \end{array}$$

where $\Pi \left({\hat{x}}^{k}\right)=H^{T}\left({\hat{x}}^{k}\right)R^{-1}H\left({\hat{x}}^{k}\right)$ is the gain matrix. The iterative process will be terminated if the $\Delta {\hat{x}}^{k}$ less than a predeterminate tolerance or the iteration step *k* is larger than a maximum number.

### 2.2. ISE Model

In this section, in order to show the impact of uncertainties on state variables more accurately, the ISE model is established. The state variables are expressed in the form of interval numbers. The settings of interval state variables are given as [23]

(6) $$\left[\hat{x}\right]=\left[\underline{\hat{x}},\bar{\hat{x}}\right]$$

where $\underline{\hat{x}}$ and $\bar{\hat{x}}$ are the lower and upper bounds of the interval state variables, respectively. Usually, $\underline{\hat{x}}<\hat{x}<\bar{\hat{x}}$.

The ISE model considering various uncertainties such as DG outputs, EV charing load demand, inaccurate line parameters. and measurement noise is described as follows. Moreover, each uncertainty is independent regardless of its correlation with the other [27]. The bounds of measurements are associated with the bounds of error, according to the setting of different uncertainties, the corresponding error setting is also different, so measurements of conventional load and generation can be depicted as

(7) $$\begin{array}{c} Z-e^{-}<Z<Z+e^{+} \end{array}$$

where $e^{+}$ and $e^{-}$ are the maximum values of the positive and negative measurement errors in *Z*.

Considering the randomness of DG outputs and EV charging load demand, the measurement formulas of DG outputs and EV charging load demand can be extended as follows. The upper and lower bounds of DG outputs and EV charging load demand are set out, respectively.

(8) $$\begin{array}{c} \left[Z_{DG1}\right]=\left[P_{DG,l},P_{DG,u}\right] \end{array}$$

(9) $$\begin{array}{c} \left[Z_{DG2}\right]=\left[Q_{DG,l},Q_{DG,u}\right] \end{array}$$

(10) $$\begin{array}{c} \left[Z_{EV1}\right]=\left[P_{EV,l},P_{EV,u}\right] \end{array}$$

where $P_{DG,l}$ and $P_{DG,u}$ represent the lower and upper bounds of the real power injection with DG outputs, respectively. $P_{EV,l}$ and $P_{EV,u}$ represent the lower and upper bounds of the real power with EV charging load demand, respectively. $Q_{DG,l}$ and $Q_{DG,u}$ represent the lower and upper bounds of the reactive power injection with DG outputs, respectively. The ISE model considers the uncertainty from measurements, the measurement vector mainly includes load demand and power outputs of DG [8] and EV charging load demand. The power outputs of DG are taken as the pseudo-measurements to achieve system observability. Although the instantaneous wind speed can be predicted more accurately, their own prediction errors will inevitably lead to greater prediction errors of DG power outputs.

Primarily, measurement data is obtained mainly through SCADA in distribution systems. SCADA can provide the data of bus voltage magnitude, branch power, and bus injection power [2]. $P_{i}$ and $Q_{i}$ are the real and reactive power injection measurement of bus *i*, respectively, and are given as [12]

(11) $$\begin{array}{ccc} P_{i} & = & V_{i}\sum_{j=1}^{m}V_{j}\left(G_{ij}cos\theta_{ij}+B_{ij}sin\theta_{ij}\right) \end{array}$$

(12) $$\begin{array}{ccc} Q_{i} & = & V_{i}\sum_{j=1}^{m}V_{j}\left(G_{ij}sin\theta_{ij}-B_{ij}cos\theta_{ij}\right) \end{array}$$

where $\theta_{ij}=\theta_{i}-\theta_{j}$. $G_{ij}$ and $B_{ij}$ represent the line conductance and the line susceptance, respectively. *i* and *j* are the bus numbers.

According to the given power factor (this paper sets the power factor to 0.95 in the subsequent calculation example verification link) [8], the conventional load and wind turbine outputs can be calculated. For the EV units [28], unit power factor control is adopted, and the reactive power demand for charging is zero. In this paper, the estimated results of adding DG units to the distribution system are obtained. As the outputs of EV units are uncertain, it is more feasible to model DG outputs and EV charging load demand to quantify the uncertainty level in interval prediction.

Considering that the dimension of measurement is lager than the dimension of state variables, the modeling and solving of the interval overdetermined equation is more difficult [8]. Therefore, the overdetermined equations are converted into a square matrix

(13) $$\begin{array}{c} {\left[\begin{array}{c} \left[H_{1}\right]\\ \left[H_{2}\right] \end{array}\right]}^{T}\left[\begin{array}{cc} W_{1} & 0\\ 0 & W_{2} \end{array}\right]\left[\begin{array}{c} \left[H_{1}\right]\\ \left[H_{2}\right] \end{array}\right]\left[\hat{x}\right]={\left[\begin{array}{c} \left[H_{1}\right]\\ \left[H_{2}\right] \end{array}\right]}^{T}\left[\begin{array}{cc} W_{1} & 0\\ 0 & W_{2} \end{array}\right]\left[\begin{array}{c} \left[Z_{1}\right]\\ {}[Z_{2}] \end{array}\right] \end{array}$$

where $\left[\hat{x}\right]$ is the interval state vector, $\left[Z_{1}\right]$ is the interval measurement vector, and $\left[Z_{2}\right]$ is the interval variables of DG power outputs and EV charging power demand. $\left[H_{1}\right]$ is the Jacobian matrix of the traditional measurements and $\left[H_{2}\right]$ is the Jacobian matrix of the DG and EV measurements. $W_{1}$ is the weighting matrix of the traditional measurements and $W_{2}$ is the weighting matrix of the DG and EV measurements.

## 3. Proposed ISE Approach

### 3.1. Proposed ISE Model

In this section, based on interval measurement model, the detailed description of various uncertainties such as measurement *z*, measurement function *H*, and error *e* are analyzed.

This paper considers the influence of the uncertainty due to the possible changes of system parameter values on the state estimation results. Considering that the influence of line parameter on the results of interval state estimation is mainly reflected in the measurement function, the extended measurement model as follows [17]:

(14) $$\begin{array}{c} z=h(x,p)+e \end{array}$$

where *p* is the system parameter vector. The Taylor expansion of this formula (only keep first-order terms and neglect higher-order terms) as follows:

(15) $$\begin{array}{ccc} z & \approx & h\left(x,p\right)+\frac{\partial h}{\partial p}▵p+e\\ & = & h(x,p)+w+e \end{array}$$

where ▵p is the deviation of system and $w=\frac{\partial h}{\partial p}▵p$. $\frac{\partial h}{\partial p}$ represents the partial derivative matrix of the measurement function with the parameter vector, expressed as $H_{p}$. Note that the model presented by Moore is a fundamental interval model including linear and nonlinear models. Considering various distribution system uncertainties, the proposed ISE model is an extension of the linear Moore model. As (14) is a nonlinear model, the first-order Taylor series expansion of the proposed model can be taken as a linear model. Besides, the uncertainties such as inaccurate line parameters and noise are also included in the nonlinear model and would be further linearized. The impacts of the DG and EV uncertainties on the model are mainly reflected in the increasing number of measurements.

Traditional state estimation is mainly based on the assumption of statistical noise. However, the pseudo-measurement of load in the distribution systems does not meet the assumption of Gaussian distribution [21]. According to the works in [29,30], the DG outputs obey non-Gaussian distribution or Gaussian mixture models. Moreover, the distribution of real-time measurement error is not always Gaussian especially due to system errors and quantization errors of acquisition equipment [9,10].

For the traditional distribution system state estimation problem, the distribution of measurement noise is usually assumed to be Gaussian. However, this assumption is only an approximation to reality [31]. One report conducted by Pacific Northwest National Laboratory reveals that PMU measurement noise is non-Gaussian [32]. The *t*-distribution with the “heavy tail” property is flexible to characterize Gaussian or non-Gaussian noise statistics [33]. That is the main reason to utilize the *t*-distribution to characterize the measurement error. The PDF of *t*-distribution is given by

(16) $$f_{i}\left(e_{i}\right)=\frac{\Gamma (\frac{v_{i}+1}{2})}{\sqrt{v_{i}\pi }\left(\xi_{i}\right)\Gamma \left(\frac{v_{i}}{2}\right)}{\left(1+\frac{|e_{i}{|}^{2}}{{\left(\xi_{i}\right)}^{2}v_{i}}\right)}^{-\frac{v_{i}+1}{2}}$$

where Γ(·) is the gamma function, $\xi_{i}$ is the scale parameter, and $v_{i}$ is the shape parameter. In addition, to the best of our knowledge, only the symmetrical distributions such as Gaussian model [34,35] and Gaussian mixture models [29] are widely used to characterize the measurement error for the distribution system state estimation. Therefore, the symmetrical distribution is applied in our proposed algorithm presented later.

### 3.2. Proposed ISE Algorithm

The MKO algorithm uses the preset value as the reference value to update iteratively during the calculation process, but the preset value is not the true value, and preset value is different from the true value after all. Using the ICP algorithm to solve the problem can ensure the convergence. The solution is more in accordance with the actual operation of the distribution system. The speed of using the ICP algorithm to obtain the solution depends to a large extent on the initial order of the equations and variables, which makes the calculation time longer, but ensures that the solution can contain a variety of uncertainties. In this subsection, a new ISE algorithm based on the MKO and ICP is proposed for DSSE to improve the ISE results. Based on (13), the linear ISE model can be further extended as AX=B.

Considering that the initial value obtained by the MKO algorithm is a preset value rather than a true value and the ICP algorithm can obtain an initial value that satisfies a variety of uncertainties, the proposed algorithm would re-select an initial value. The inverse of the midpoint matrix of *A* is used as the preprocessing factor $M={\left(Mid\left[A\right]\right)}^{-1}$. The midpoint variables of *B* times *M* to obtain an approximate solution $X_{s}$, that is, $X_{s}=\left(Mid\left[B\right]\right)\times M$.

Starting from the initial preset value $X^{\left(0\right)}$ by (17).

(17) $$\begin{array}{c} X^{\left(0\right)}={\left(\left[-\frac{{∥MB∥}_{\infty }}{{1-∥E-MA∥}_{\infty }},\frac{{∥MB∥}_{\infty }}{{1-∥E-MA∥}_{\infty }}\right],...,\left[-\frac{{∥MB∥}_{\infty }}{{1-∥E-MA∥}_{\infty }},\frac{{∥MB∥}_{\infty }}{{1-∥E-MA∥}_{\infty }}\right]\right)}^{T} \end{array}$$

where *M* satisfies ∥E−MA∥<1 and the E is the identity matrix.Various uncertainties are introduced as constraints to make the final solution closer to the true value. Considering variables $X_{i}$ and a constraint *c*, the constraint equation is given as

(18) $$\begin{array}{c} d(X_{i})=0 \end{array}$$

(19) $$\begin{array}{c} X_{i}^{c}=g^{c}\left(X_{i}\right) \end{array}$$

where *d* is measurement function and $g^{c}$ is constraint function related to state vector. $X_{i}^{c}$ is the value obtained by constraint function. Based on the Equations (18) and (19), the initial value $I(X^{\left(0\right)})$ can be obtained when considering the uncertainty constraints. Then, according to $X^{\left(0\right)}$ and $I(X^{\left(0\right)})$, the suitable initial value is given as

(20) $$\begin{array}{c} X_{New}^{\left(0\right)}=I\left(X^{\left(0\right)}\right)\cap X^{\left(0\right)} \end{array}$$

By modifying the difference between the value $X_{New}$ of the *k*-th iteration and the approximate solution $X_{s}$, the result is guaranteed to be sufficiently compact. Based on formula AX=B, an interval vector *u* can be calculated by

(21) $$\begin{array}{c} A^{-1}B=MB+\left(E-MA\right)A^{-1}B\in MB+\left(E-MA\right)u \end{array}$$

Defining $l=X_{New}-X_{s}$ and getting an interval equation $Al=B-AX_{s}$, the initial equation of $l^{\left(0\right)}$ is $l^{\left(0\right)}=X_{New}^{\left(0\right)}-X_{s}$ and the enclosure $l^{\left(k+1\right)}$ is given as

(22) $$\begin{array}{c} l^{\left(k+1\right)}=\left(M\left(B-AX_{s}\right)+\left(E-MA\right)l^{\left(k\right)}\right)\cap l^{\left(k\right)} \end{array}$$

until $∥l^{\left(k+1\right)}-l^{\left(k\right)}{∥}_{\infty }\leq \varepsilon$. The *k*-th solution

(23) $$\begin{array}{ccc} X_{New}^{\left(k\right)} & = & X_{s}+l^{\left(k\right)}\\ & ⊇ & \left(MB+\left(E-MA\right)X_{New}^{\left(k\right)}\right)\cap X_{New}^{\left(k\right)} \end{array}$$

Then, use the $X_{New}^{\left(k\right)}$ to calculate the *k*-th result $X_{f}^{\left(k\right)}$, the equation gives as

(24) $$\begin{array}{c} X_{f}^{\left(k\right)}=g^{c}\left(X_{New}^{\left(k\right)}\right)\cap X_{New}^{\left(k\right)} \end{array}$$

Then, according to the Equation (25), stop the iteration when the sum of the width of the interval state estimation results reduces to the convergence criterion. The convergence criterion is the Formula (26). If the result does not satisfy the convergence criterion, $X_{f}^{\left(k\right)}$ is required to be recalculated.

(25) $$\begin{array}{c} r\left(k\right)=(\sum_{k=1}^{n}\left(Wid\left(X_{f}^{\left(k+1\right)}\right)-Wid\left(X_{f}^{\left(k\right)}\right)\right))/n \end{array}$$

(26) $$\begin{array}{c} r(k)<\varepsilon \end{array}$$

where the $Wid(X_{f}^{\left(k+1\right)})$ is the sum of the width of the (k+1)-th interval state estimation results. If not convergent, $X_{f}^{\left(k\right)}$ is used as the initial value $X_{New}^{\left(0\right)}$ to recalculate.

**Figure 1:**
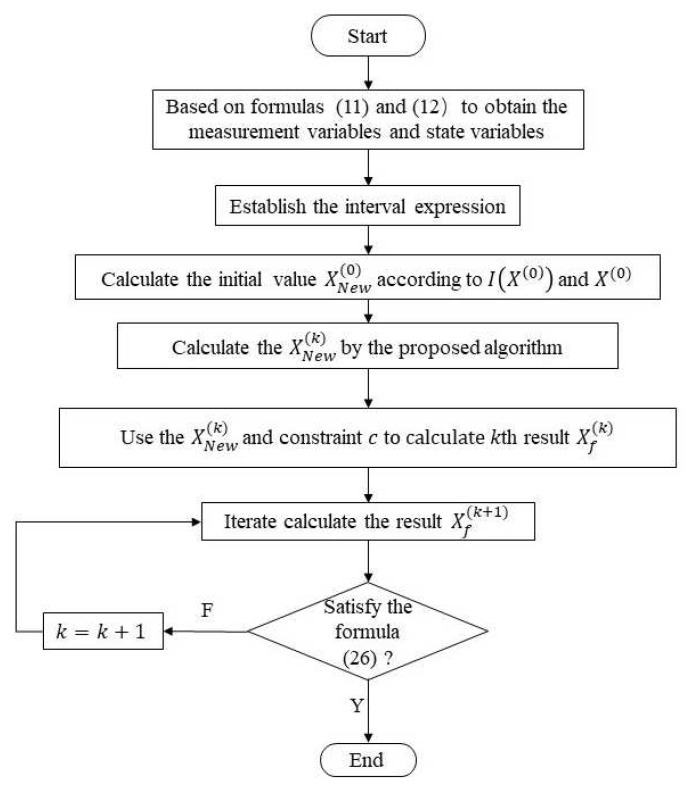
Flow chart of the proposed interval state estimation algorithm.

The difference between the proposed method and the traditional ISE methods mainly lies in the following points. First, the result of the traditional ISE methods depend largely on the choice of initial value. The MKO algorithm uses the preset value as the reference value to update iteratively during the calculation process. Note that the preset value is different from the true value. The ICP method depends heavily on the setting of initial value. Therefore, the proposed method combines the two methods to select a more appropriate initial value to reduce the conservatism of state estimation result. Besides, the proposed method ensures that the *k*-th results are close to the true value, and there will be no overall deviation of the interval results. Thus, the (k+1)-th interval results can still include the true value. Combining the proposed algorithm and ISE model together, the proposed algorithm has a certain improvement in calculation accuracy and the calculated results are more accurate. The flow chart is shown in Figure 1. The steps of the interval state estimation with the proposed algorithm considering multiple uncertainties as shown in the flowchart.

According to the work in [27], the reduction of the interval is due to the reductive interval arithmetic. Note that the arithmetic can be implemented when the correlation between uncertainties is known. However, the strong randomness of DG outputs and EV charing load demand makes it difficult to describe their behaviors accurately [20,24] so that the interval state estimation is necessary. In addition, the DG and EV applied in this paper belong to different individuals and they do not affect each other. Thus, their parameters about the uncertainties are uncorrelated. Therefore, our proposed algorithm is implemented on the fact that the parameters of DG and EV are uncorrelated.

## 4. Simulation and Results

**Figure 2:**
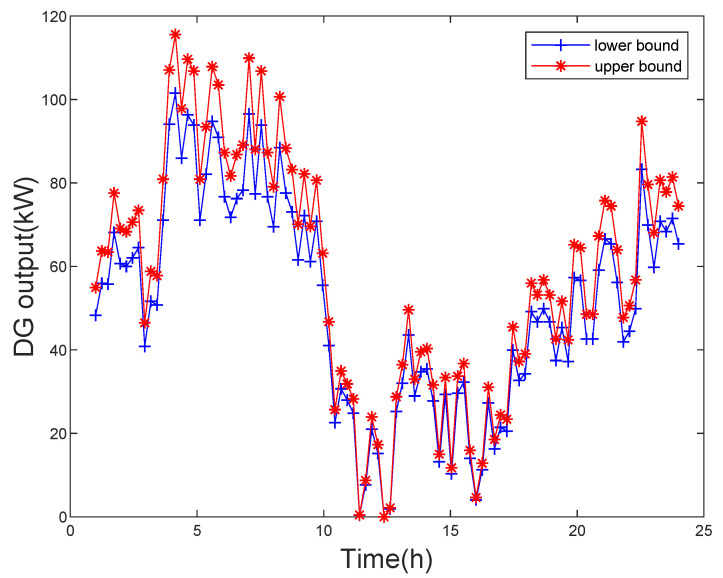
DG outputs of one day.

The simulations are performed on the IEEE 33-bus, 69-bus, and 123-bus distribution systems. Different DG outputs and EV charging demand have different effects on all tests. Therefore, a model containing different DG outputs, EV charging load demand, and constant line parameters is established. The measurement values are calculated by the power flow program. The data setting criteria for simulation are given here. The active and reactive powers at conventional load buses and EV load bus are obtained by the power flow program. The measurements are composed of truth value, line parameter influence value and the measurement error. The line parameter influence value can be calculated by the method in [17]. Assume that the maximum deviation of line parameters from the nominal value is 10%, and it is a uniform distribution. Then, the interval measurement values obtained by addition or subtraction errors. This paper chooses the *t*-distribution noises that the $v_{i}$ degree of freedom is 2 and $\xi_{i}$ is 0.005. The tolerance ε is set as $10^{-4}$. The errors of voltage bus magnitude and phase angle are set as 0.005 and $10^{-4}$, respectively. Besides, The output curve of DG unit is shown in the Figure 2. It is clear that the active power output of DG unit varies in one day. The reactive power output of DG unit can be calculated by the phase angle, where the phase angle lag is set to 0.95. The simulations are performed in MATLAB 2010b using an Intel-i7 3.19 GHz desktop with 16 GB of RAM memory computer and the INTLAB package is used for interval computation.

### 4.1. Result Analysis

In this subsection, the average width of the voltage magnitude $W_{1}$, the minimum width of the voltage magnitude $W_{2}$ and the maximum width of the voltage magnitude $W_{3}$ are utilized to measure the quality of the state estimation results according to [8,25]

(27) $$\begin{array}{ccc} W_{1} & = & \frac{1}{n}\sum_{i=1}^{n}\left(\bar{x_{i}}-\underline{x_{i}}\right) \end{array}$$

(28) $$\begin{array}{ccc} W_{2} & = & \min_{i=1,2,...,n}\left(\bar{x_{i}}-\underline{x_{i}}\right) \end{array}$$

(29) $$\begin{array}{ccc} W_{3} & = & \max_{i=1,2,...,n}\left(\bar{x_{i}}-\underline{x_{i}}\right) \end{array}$$

where *n* is the number of state estimation variables, and $\bar{x_{i}}$ and $\underline{x_{i}}$ are the upper and lower bounds of *i*-th state variable, respectively. The smaller the value of $W_{1}$ indicates that the upper and lower bounds of the state estimation result are closer to the true value.

**Figure 3:**
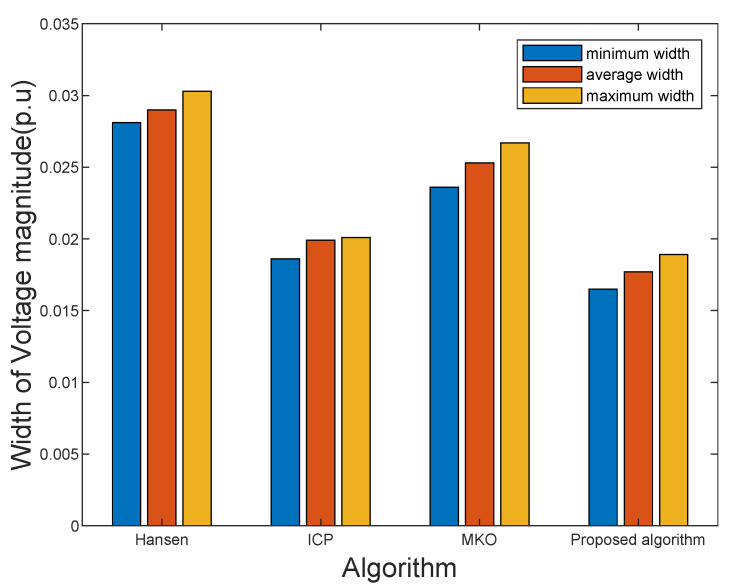
Comparison of bus voltage magnitude values in interval state estimation results on the IEEE 33 bus system.

**Figure 4:**
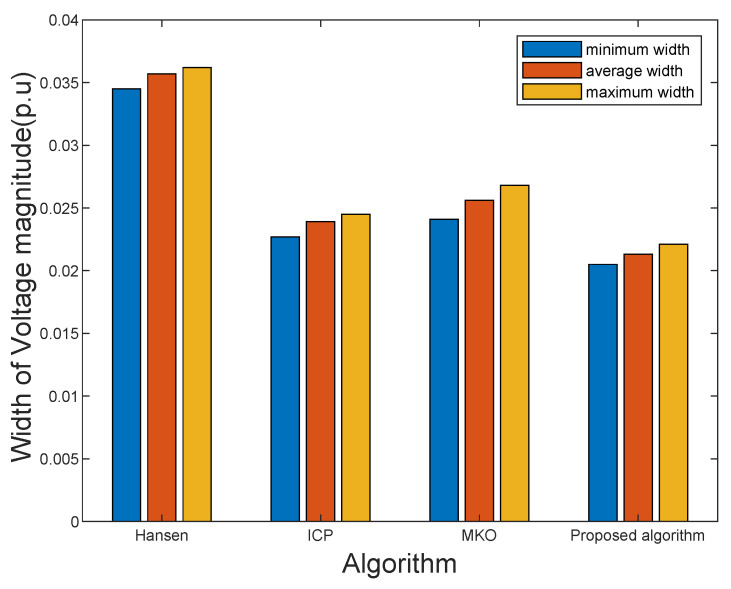
Comparison of bus voltage magnitude values in interval state estimation results on the IEEE 123 bus system.

The simulation results obtained by the proposed ISE algorithm and other existing approaches are given in Table 1. When multiple uncertainties are considering, the proposed algorithm has achieved better results than other algorithms in the IEEE 33-bus, 69-bus, and 123-bus distribution systems. Concretely, the $W_{1}$ of the three systems obtained by the proposed algorithm are 0.0177, 0.0189, and 0.0213, respectively, and they are the smallest in different systems. The results obtained by the proposed algorithm are better than the ones obtained by other approaches. For example, compared with the MKO algorithm, the proposed algorithm at least has a 9% improvement. Taking the IEEE 33-bus and 123-bus distribution systems as the illustrative example, as shown in Figure 3 and Figure 4, it is significant that the proposed algorithm can obtain tighter upper and lower bounds of state estimation variables than the Hansen, ICP, and MKO approaches. As shown in Figure 3, the maximum width of voltage magnitude obtained by the proposed algorithm and MKO are 0.0201 and 0.0267 respectively. The minimum width of voltage magnitude obtained by the proposed algorithm and MKO are 0.0186 and 0.0236, respectively.

**Figure 5:**
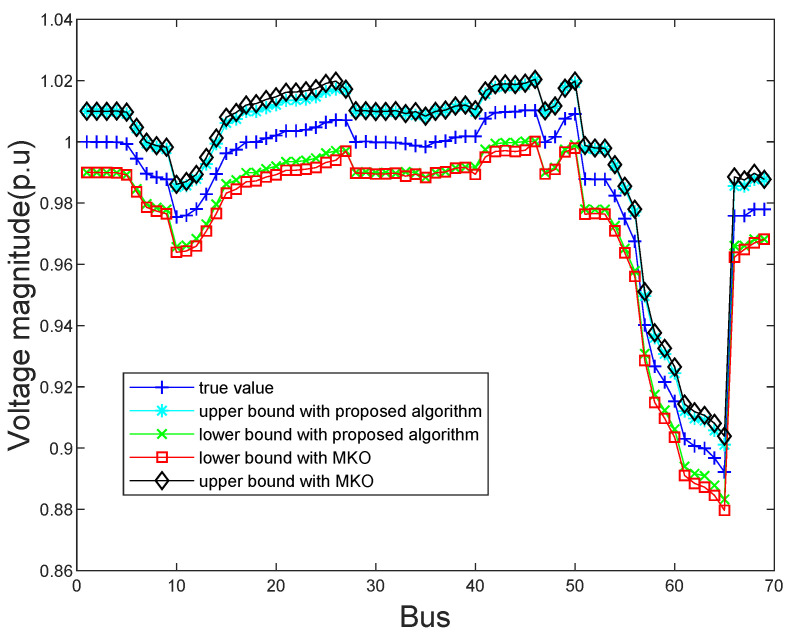
The estimated bus voltage magnitudes obtained by the proposed algorithm and MKO on the IEEE 69 bus system.

**Figure 6:**
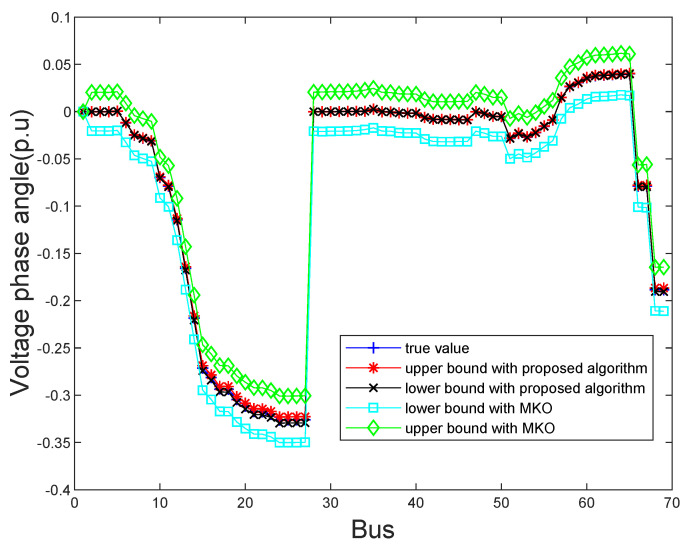
The estimated bus voltage phase angles obtained by the proposed algorithm and MKO on the IEEE 69 bus system.

In order to show the exact state estimation results obtained by the proposed algorithm and the MKO algorithm more intuitively, the estimated bus voltage magnitudes and phase angles on IEEE 69-bus system are shown in Figure 5 and Figure 6. It is clear that the upper and lower bounds of the state estimation results of two algorithms can contain true values. However, the proposed algorithm can obtain more tighter bounds of state estimation results than the MKO approach. Even though a variety of uncertainties occur, our proposed algorithm can still make the state estimation results within the normal operating range and that demonstrates that our proposed algorithm is reliable.

### 4.2. Effect of DG Units Uncertainty

In this section, the effectiveness of the proposed algorithm is verified when different numbers of DG units are introduced into the IEEE 33-bus system. DG has many forms such as wind turbine generators (WTGs) and photovoltaic (PV) panels. The DG units introduced into the cases in this paper are all WTG units. The instability and uncertainty of the weather make the output of WTGs have strong uncertainty. The modified IEEE 33-bus system in this section is based on the original IEEE 33-bus system, where DG units are added to different buses. Table 2 displays the serial number of the access bus when different DG units are connected to the system. The voltage magnitude and phase angle of the bus also fluctuate within a certain range due to the interval fluctuation of the DG units added in the system will improve the system voltage level to a certain extent.

**Figure 7:**
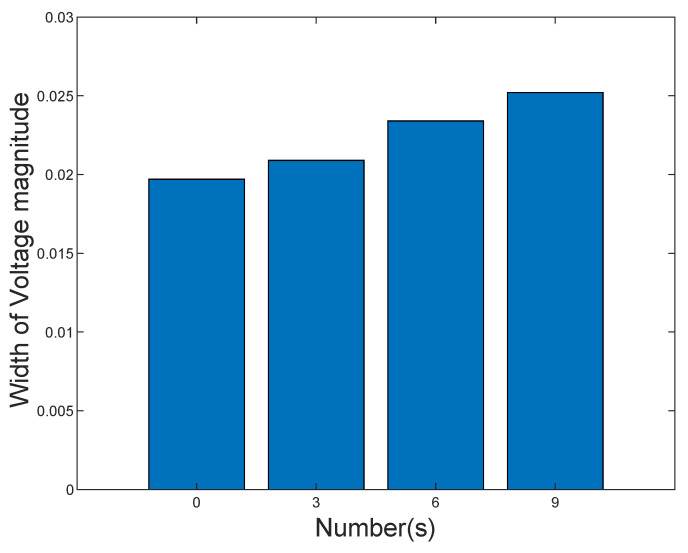
$W_{1}$ of voltage magnitude with different DG units.

The number of DG units connected to distribution systems has different effects on the operation status of the distribution system. Figure 7 illustrates the influence of the different numbers of DG units on the state estimation results. The $W_{1}$ of voltage magnitude increases when the number of DG units increases. The main reason is that more uncertainties are included in the ISE model. However, the $W_{1}$ is maintained in a small range, which indicates that the proposed algorithm can effectively alleviate the conservatism.

### 4.3. Effect of EV Units Uncertainty

**Figure 8:**
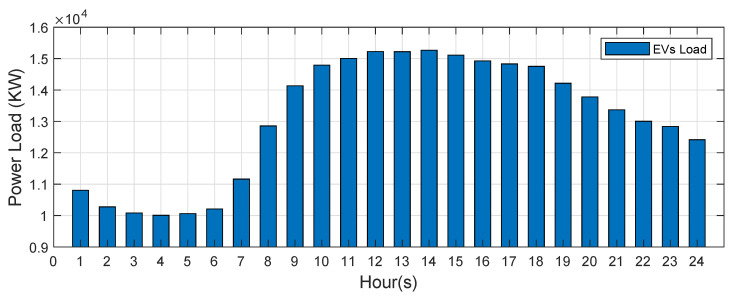
The EV units power load of one day.

**Figure 9:**
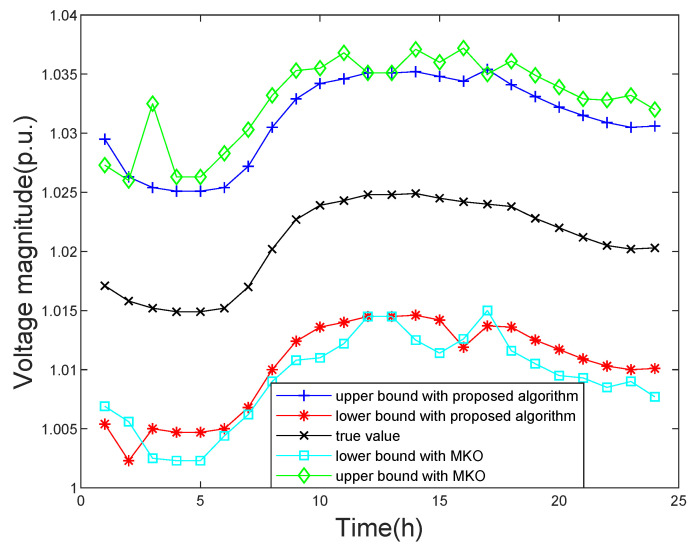
The daily variation of bus voltage magnitude consider EV units.

In order to study the influence of the uncertainty of EV charging load to the ISE, this paper uses the Monte Carlo sampling method based on statistical data laws and interval numbers to predict the EV charging load demand according to [28]. Figure 8 shows the load changes of electric vehicles in one day. In order to show the state estimation results obtained by the proposed algorithm and the MKO algorithm more intuitively, the state estimation results obtained by the two algorithms are compared on the IEEE 69-bus system. This IEEE 69-bus system connects electric vehicles at bus 69 and record the state estimation results change at bus 68. The state estimation results at bus 68 obtained through the proposed algorithm and MKO algorithm are shown in Figure 9. The proposed algorithm can obtain tighter bounds of estimation results than the MKO. In addition, the $W_{1}$ obtained by the proposed algorithm is still better than MKO, as given in Table 1. This verifies the proposed algorithm can effectively alleviate the conservatism of the uncertain state estimation solution, eliminating the interval state estimation of actual calculation cannot exist.

### 4.4. Calculation Efficiency

The following is a comparative analysis of the calculation efficiency of the six algorithms, and the statistical results are as shown in Table 3. Even though the calculation time of the proposed algorithm is a little larger than other existing methods, the proposed algorithm can obtain tighter bounds of state estimation results according to Table 1 and the state estimation results remain stably under a variety of uncertainties. Moreover, the typical measurement sampling interval is 2 to 4 s while the estimates are usually updated only once every few minutes [35,36,37]. Therefore, the computational time of our proposed algorithm is acceptable in practice.

## 5. Conclusions

In this paper, an interval state estimation model containing multiple uncertainties consisting of the non-Gaussian measurement noise, the inaccurate line parameters, the stochastic power outputs of distributed generations (DG), and the plug-in electric vehicles (EV) is established. Moreover, a new ISE algorithm based on the MKO and ICP is proposed for distribution systems so as to obtain the state estimation results with less conservativeness. Simulation results on the IEEE 33-bus, 69-bus, and 123-bus distribution systems demonstrate that our proposed ISE algorithm can obtain tighter bounds of state estimation variables than other common approaches such as the KM, ICP, KM-ICP, Hansen, and MKO.

## Figures and Tables

**Table 1:** The average width of voltage magnitudes for the three systems.

System	Hansen [23]	Krawczyk [24]	ICP [14]	KM-ICP [25]	MKO [8]	Proposed Algorithm
IEEE33	0.029	0.0282	0.0199	0.0192	0.0253	0.0177
Improvement	38.9	37.2	30.03	7.81	11.05	0
IEEE69	0.0276	0.0261	0.0209	0.0203	0.0227	0.0189
Improvement	31.52	27.58	16.7	6.89	9.56	0
IEEE123	0.0357	0.0354	0.0239	0.0235	0.0256	0.0213
Improvement	40.4	39.8	18.9	10.6	12.4	0

Proposed algorithm result’s improvement (%) = $\frac{\left(\mathrm{Other}\;\mathrm{algorithm}-\mathrm{Proposed}\;\mathrm{algorithm}\right)}{\mathrm{Other}\;\mathrm{algorithm}}\times 100$. Proposed algorithm result’s improvement over itself is 0%.

**Table 2:** The bus with DG unit in the IEEE 33-bus system.

Number	Bus Node
0	-
3	21,24,32
6	14,19,24,27,32,33
9	2,3,14,19,21,24,27,32,33

**Table 3:** The calculation efficiency for the three systems.

System	Hansen [23]	Krawczyk [24]	ICP [14]	KM-ICP [25]	MKO [8]	Proposed Algorithm
IEEE33	0.1146	0.1247	0.2018	0.1976	0.1672	0.3025
IEEE69	0.2457	0.2488	1.0279	0.2319	1.3671	1.6142
IEEE123	14.3678	14.7462	27.623	12.9574	29.373	32.33

The calculation time unit of each algorithm: second.
